# Isolation, Identification, Drug Sensitivity, Pathogenicity and Genomic Analysis of *Vibrio mimicus* from *Pelteobagrus fulvidraco*

**DOI:** 10.3390/microorganisms14081842

**Published:** 2026-08-19

**Authors:** Shao Peng, Kai Zhang, Zichun Zhou, Zhi Meng, Chunyu Zhang, Wenjing Li, Tongpu Guo, Lei Tang, Wei Xu, Wenli Zhou, Xuying Jia, Jinwei Gao

**Affiliations:** 1 School of Life Sciences, Tianjin University, Tianjin 300072, China; 2 Tianjin Key Laboratory of Fishery Resources and Environmental Ecology, College of Fisheries, Tianjin Agricultural University, Tianjin 300384, Chinasaz0908@126.com (W.Z.); jiaxuying@tjau.edu.cn (X.J.); gaojinwei@tjau.edu.cn (J.G.)

**Keywords:** *Pelteobagrus fulvidraco*, *Vibrio mimicus*, virulence factor, drug resistance gene, median lethal dose

## Abstract

In this study, pathogenic bacteria were isolated from diseased *Pelteobagrus fulvidraco* collected from a yellow catfish farm in northern China. The recovered isolate was subjected to Gram staining, transmission electron microscopy observation, *16S* rRNA gene sequencing and whole-genome sequencing, followed by systematic analyses of its antimicrobial resistance, pathogenicity and genomic characteristics. Combined with artificial infection assays and multiple comparative verifications, the pathogen was identified as *Vibrio mimicus*. Clinical observation of naturally infected fish revealed that superficial skin ulcers of varying severity constituted the hallmark clinical sign of this disease. Genomic analysis uncovered major virulence-related genes, including *tlh*, *vmh* and *tdh*, as well as key antimicrobial resistance genes such as *crp* and *ugd*. Artificial infection trials confirmed the strong virulence of this isolate against *P. fulvidraco*, with distinct clinical manifestations triggered by injections of bacterial suspensions at different concentrations. Mortality was first observed in the high-dose group at 2–3 days post-injection, yet no cutaneous ulceration was detected at this stage. The peak incidence and mortality occurred between 3 and 7 days post-challenge, with diseased fish exhibiting lesions identical to those seen in naturally infected individuals. The median lethal dose (LD_50_) of the isolate was determined to be 1.72 × 10^5^ CFU/mL. Growth gradient assays across varying temperatures and pH values identified the optimal culture conditions for this strain: 33 °C and pH 8.5. Antimicrobial susceptibility testing demonstrated that the isolate was highly susceptible to piperacillin, norfloxacin, roxithromycin, azithromycin, furazolidone, ceftriaxone and tetracycline, whereas it displayed high-level resistance to compound sulfamethoxazole and clindamycin. Collectively, this work lays a scientific foundation for the isolation, identification and comprehensive research of *Vibrio mimicus*-induced ulcerative disease in yellow catfish.

## 1. Introduction

The yellow catfish *(Pelteobagrus fulvidraco)* is a commercially valuable freshwater teleost belonging to the class Osteichthyes, order *Siluriformes*, family *Bagridae* and genus *Pelteobagrus*. This species is widely cultivated across Southeast Asia, with China as its primary farming country [1]. As an emerging high-value aquaculture species in China, its national total output reached 599,800 tons in 2022 [2]. Nevertheless, intensive high-density farming has triggered frequent outbreaks of bacterial diseases in farmed yellow catfish in recent years. A prevalent, highly lethal ulcerative syndrome has become one of the most destructive diseases threatening domestic yellow catfish aquaculture, characterized by severe square-shaped skin ulcers distributed on the head, dorsum and caudal peduncle, thus commonly termed “square ulcer disease”. Bacteria are the dominant causative agents of fish skin ulceration; well-documented pathogenic strains include *Vibrio anguillarum*, *Vibrio vulnificus*, *Aeromonas salmonicida* and *Aeromonas veronii* biovar *sobria* [3]. Since 2011, successive large-scale epidemics of this ulcerative disease have broken out in yellow catfish farms nationwide, and pathogenic identification has confirmed the pathogen as *Vibrio mimicus*, which can induce cumulative mortality ranging from 70% to 100% in infected fish [4,5]. Recent multi-omics and immunological studies further verified that *Vibrio mimicus* specifically invades the epidermal and muscular tissues of yellow catfish, triggering a severe inflammatory storm and tissue remodeling, which directly lead to typical square ulcer lesions [6,7]. Additionally, genome-wide association analysis identified multiple SNP loci and immune-related candidate genes tightly linked to host resistance against *Vibrio mimicus*, providing novel molecular evidence for host–pathogen interaction mechanisms [8].

*Vibrio mimicus* is a Gram-negative, slightly curved short rod belonging to the family *Vibrionaceae*, genus *Vibrio*, featuring polar flagella but no capsules or endospores. Taxonomically, vibrios are classified into four groups: *Vibrio cholerae O1*, atypical *Vibrio cholerae O1*, *non-O1 Vibrio cholerae* and other vibrio species. The *non-O1 Vibrio cholerae* cluster covers major aquatic pathogens such as *Vibrio parahaemolyticus*, *Vibrio mimicus*, *Vibrio alginolyticus* and *Vibrio vulnificus* [9]. Ubiquitously distributed in all types of aquatic ecosystems, *Vibrio mimicus* has seen a sharp rise in infection prevalence alongside the rapid expansion of global intensive aquaculture, resulting in enormous economic losses for the aquaculture sector. This bacterium is recognized as both a foodborne human pathogen and a critical zoonotic agent shared by humans and aquatic animals [10]. In human clinical cases, ingestion of contaminated seafood carrying *Vibrio mimicus* frequently causes acute gastroenteritis, watery diarrhea and food poisoning [11,12,13,14] Natural *Vibrio mimicus* infections have also been reported in a broad spectrum of aquatic hosts, including red swamp crayfish *Procambarus clarkii* [15], Australian freshwater crayfish *Cherax quadricarinatus* [16], and gibel carp *Carassius gibelio*, in which the pathogen induces peri-branchial and peri-orbital congestion, swollen anus and abdominal ascites [17]. Environmental parameters such as ambient pH significantly modulate the expression of virulence phenotypes and extracellular toxic products of *Vibrio mimicus*, further accelerating disease outbreaks under suboptimal culture water quality [18].

Although previous studies have advanced our understanding of the pathological characteristics and host immune responses of *Pelteobagrus fulvidraco* infected with *Vibrio mimicus*, several critical research gaps remain. Most current investigations focus predominantly on omics screening and macroscopic phenotypic observation, while the systematic phenotypic, pathogenic, and genomic profiles of epidemic *Vibrio mimicus* strains prevalent in northern China remain poorly characterized. Accordingly, the present study provides a fundamental scientific basis for the isolation, identification, and comprehensive characterization of ulcerative disease caused by *Vibrio mimicus* in farmed yellow catfish.

## 2. Materials and Methods

### 2.1. Experimental Design

**Figure 1:**
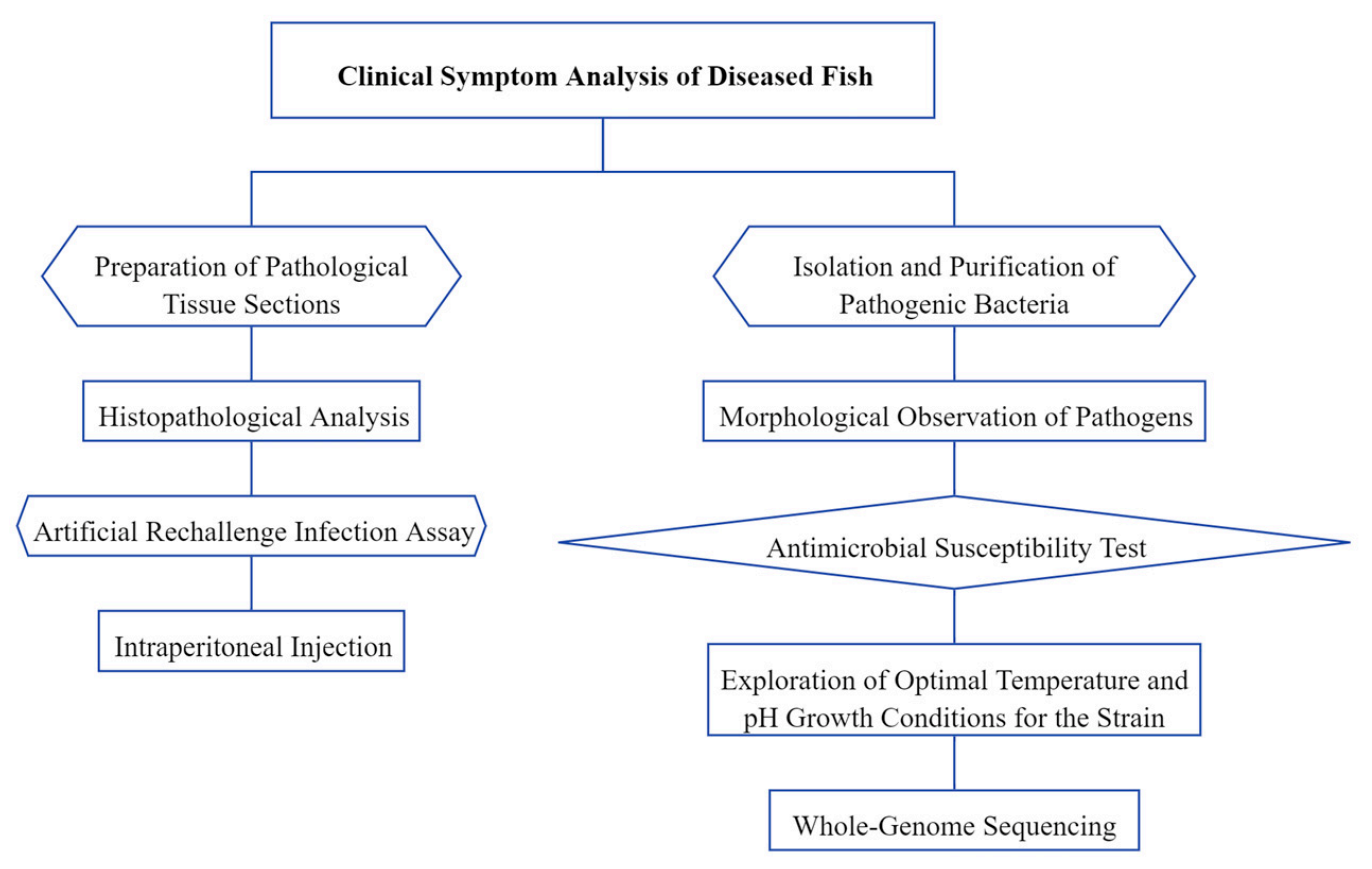
Technical roadmap.

This study adopted a comprehensive multi-dimensional integrated research design combining field sampling, phenotypic observation, pathogen identification, artificial infection challenge, environmental growth characterization, whole-genome sequencing and antimicrobial susceptibility detection. The technical roadmap of this study is shown as follows (Figure 1).

### 2.2. Experimental Materials

In June 2025, a large number of diseased fish appeared in a yellow catfish pond farm in North China, and the mortality rate increased suddenly. The severe disease outbreak here results in substantial economic losses, so this epidemic was selected as a typical research case. We collected 5 diseased *Pelteobagrus fulvidraco* with obvious surface ulceration symptoms, transferred them to the key laboratory of Tianjin Agricultural University, and took photos to record the ulceration symptoms and occurrence of diseased fish. (The key laboratory is a university-affiliated aquatic animal disease diagnostic platform equipped with professional pathogen-detection instruments for bacterial isolation, molecular identification and aquaculture-related basic research).

### 2.3. Clinical Observation of Diseased Fish

Diagnosis was performed using clinical diagnostic methods for aquatic animals. Prior to sampling, the body weight and body length of experimental fish were recorded using a ruler and electronic balance. The diseased fish were necropsied under aseptic conditions, with the abdominal cavity dissected for visual assessment of pathological lesions in internal tissues and organs.

### 2.4. Pathological Tissue Section

Collect muscle, kidney, intestine, liver, and spleen tissues from diseased fish and prepare paraffin sections. Fix the tissues with 4% paraformaldehyde, then trim the tissues, perform gradient dehydration, clearing, paraffin embedding, wax trimming, transverse and longitudinal sectioning and other operations. Stain the histopathological sections with hematoxylin-eosin (H&E), mount them with neutral gum and perform optical microscopy examination and analysis.

### 2.5. Isolation, Purification and Morphological Observation of Pathogenic Bacteria

Under sterile conditions, we used a sterile inoculating loop to sample lesions on the body surface, as well as muscle, intestine, spleen, kidney and liver tissues from diseased fish. Samples were streaked onto TSA medium using the three-streak isolation method, followed by incubation at 30 °C in a constant-temperature incubator. After 24 h of incubation, a single dominant colony was picked from the TSA plate and streaked onto TCBS nutrient agar for preliminary screening of Vibrio strains. The selected colonies were sub-cultured repeatedly via multiple rounds of streak purification to obtain pure isolates, which were subsequently preserved. Colony morphology was observed macroscopically. Bacterial morphological features were examined by light microscopy following Gram staining and further characterized using transmission electron microscopy (This experiment was completed in collaboration with Sangon Biotech (Shanghai) Co., Ltd., Shanghai, China).

### 2.6. Artificial Reinfection Test

Healthy yellow catfish (body weight, 120 ± 12.5 g) were acclimated in a recirculating aquaculture system at 20 ± 1 °C for one week and then randomly allocated to six groups (*n* = 10 per group). Six experimental water tanks were prepared, with 10 experimental fish reared in each tank. The temperature was optimal for rearing *Pelteobagrus fulvidraco*. Artificial infection was performed by intraperitoneal injection. The challenge strain was cultured in LB medium with shaking at 120 r/min and 28 °C for 24 h. The bacterium was cultured at the optimal temperature for pathogen assays. Bacterial cells were harvested by centrifugation at 5000 rpm for 5 min and resuspended in sterile phosphate-buffered saline (PBS) to obtain the stock bacterial suspension. This suspension was ten-fold serially diluted with PBS to generate working suspensions at concentrations of 1.5 × 10^8^, 1.5 × 10^7^, 1.5 × 10^6^, 1.5 × 10^5^, 1.5 × 10^4^, and 1.5 × 10^3^ CFU/mL. Each fish received an intraperitoneal injection of 0.2 mL bacterial suspension; the fish in the blank-control group were injected with 0.2 mL sterile PBS instead. Disease incidence and mortality were recorded daily over a 10-day continuous observation period. Probit analysis implemented in SPSS 27.0 was applied to determine the LD_50_ value of the challenge strain.

### 2.7. 16SrDNA Gene Sequencing and Phylogenetic Tree Analysis

Bacterial DNA was extracted using the Aquatic Animal Pathogen Gene DNA Extraction Kit (FAST) as the amplification template. The amplification of target genes was carried out using universal primers and specific primers designed based on the vmh virulence gene of *Vibrio mimicus*, respectively, according to references (Table 1) [19]. Amplification conditions: 95 °C pre-denaturation for 2.5 min; 95 °C denaturation for 15 s; 55 °C annealing for 30 s; 72 °C extension for 60 s; 35 cycles in total; extension at 72 °C for 10 min. The reaction system consisted of 50 μL, with 25 μL of PCR mix, 1.5 μL of each of the upper and lower primers, and 2 μL of template, supplemented with ddH_2_O. Take 5 μL of PCR product and use 1% agarose gel electrophoresis to determine whether it is a clear single band. The PCR product was sequenced by Sangon Biotech. The *16S* rRNA sequences obtained from sequencing were aligned with known nucleic acid sequences in GenBank. Using MEGA 11 software, multiple sequence alignments were performed on the strains with high sequence similarity obtained from the GenBank database using the ClustalW algorithm, and conservative sites were manually corrected. The phylogenetic tree was constructed using the neighbor-joining method and tested using the bootstrap method (with 1000 repetitions).

### 2.8. Exploring the Influence of Temperature and pH on the Proliferation of Vibrio mimicus

#### 2.8.1. The Impact of Temperature on the Proliferation of *Vibrio mimicus*

To investigate the effect of temperature on the growth of *Vibrio mimicus*, the controlled variable method was employed. Based on the results of preliminary experiments, temperature groups of 18 °C, 23 °C, 28 °C and 33 °C were selected. For each group, 500 μL of bacterial solution was aspirated at a ratio of 1% (*v*/*v*) and added to 50 mL of LB liquid medium with pH7.0. The cultures were then incubated at different temperatures with shaking at 120 r/min for 24 h. The absorbance values at OD600 were measured using a UV spectrophotometer (Beijing Purkinje General Instrument Co., Ltd., Beijing, China), and growth curves were plotted.

#### 2.8.2. The Effect of pH on the Proliferation of *Vibrio mimicus*

To investigate the effect of pH on the growth of *Vibrio mimicus*, pH conditions of 3.0, 4.0, 5.0, 6.0, 7.0, 8.0, 8.5, 9.0 and 10.0 were selected. The pH of the LB liquid medium was adjusted to these values using HCl and NaOH. For each group, 500 μL of bacterial solution was aspirated at a ratio of 1% (*v*/*v*) and added to 50 mL of LB liquid medium with different pH values. The cultures were then incubated at 120 r/min for 24 h under different pH conditions. The absorbance values at OD600 were measured using a UV spectrophotometer (Beijing Purkinje General Instrument Co., Ltd., Beijing, China), and growth curves were plotted.

### 2.9. Genome Sequencing

This experiment was conducted by Shanghai Sangon Biotech Co., Ltd. (Shanghai, China). For functional gene annotation, protein-coding genes were predicted via Prodigal, and gene function was annotated against the NR, COG, KEGG, VFDB virulence factor database and CARD antibiotic resistance database by NCBI-BLAST (https://www.ncbi.nlm.nih.gov/ accessed on 30 July 2025) alignment.

### 2.10. Antimicrobial Susceptibility Testing

The tested antimicrobial drugs encompass 15 types across 9 major categories, including 3 types of quinolones: levofloxacin (CIP), norfloxacin and ofloxacin; aminoglycosides: kanamycin and gentamicin; cephalosporins: cefotaxime and cefoperazone; macrolides: roxithromycin and azithromycin; penicillins: penicillin and piperacillin; sulfonamides: sulfamethoxazole; nitrofurazones: furazolidone; tetracyclines: tetracycline; lincomycin: clindamycin. Inoculate pathogenic bacteria into liquid medium, incubate with shaking at 28 °C for 24 h, adjust the concentration of the bacterial solution to an OD value of 1 × 10^8^ CFU/mL, take 100 microliters of pure bacterial solution and evenly spread it on a plate. Using the Kirby–Bauer disk diffusion method, place 15 antimicrobial drug disks on the plate and incubate at 28 °C for 24 h. Record the size of the inhibition zone of the antimicrobial susceptibility disk and evaluate the results according to the antimicrobial susceptibility testing standards of the Clinical and Laboratory Standards Institute.

## 3. Results and Analysis

### 3.1. Clinical Symptom Analysis of Diseased Fish

**Figure 2:**
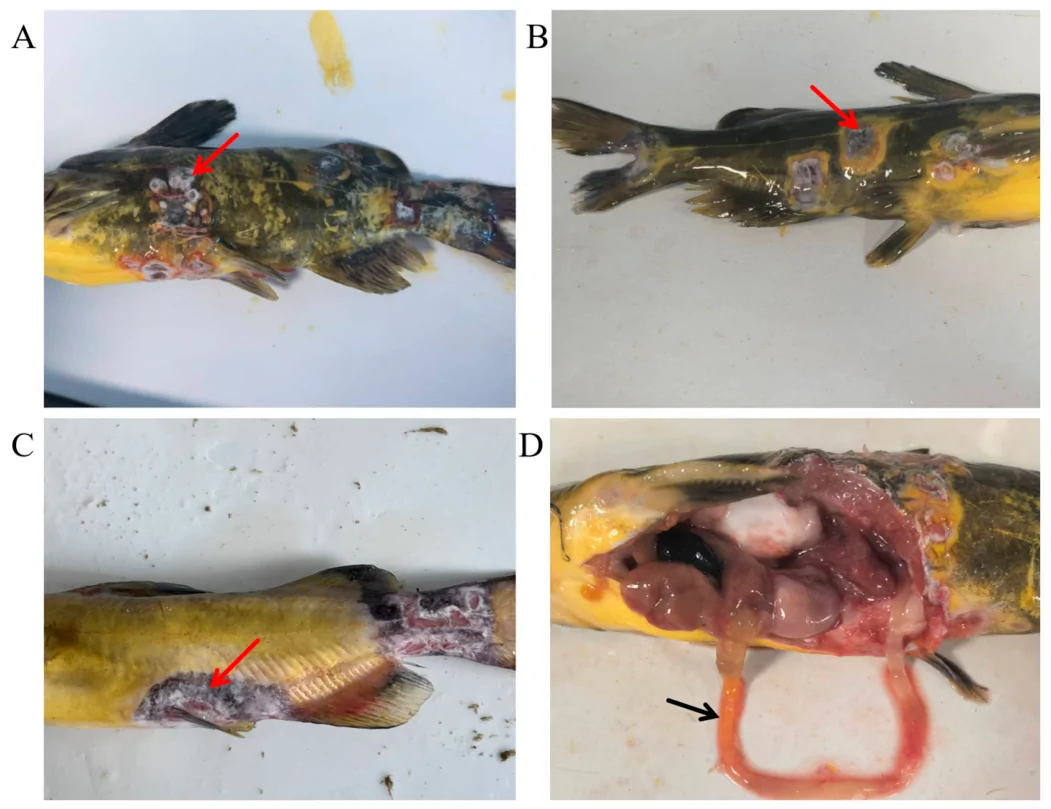
The body surface and internal organs of the diseased fish. (**A**) Images of skin clinical signs in diseased fish; the ulcer lesions appeared punctate, as indicated by the red arrows. (**B**) Images of skin clinical signs in diseased fish; the ulcer foci were square in shape, as indicated by the red arrows. (**C**) Large areas of ulcer lesions were visible on the skin of diseased fish, as indicated by the red arrows. (**D**) Photograph of the abdominal cavity of diseased fish at necropsy; the black arrow indicates that the intestines are red and swollen, with effusion.

Large square ulcer lesions appeared on the fish skin. As shown in the figures, the clinical lesions exhibited varying severities: minor focal lesions (Figure 2A), small ulcerated areas (Figure 2B) and extensive severe ulceration (Figure 2C). Severe muscular hemorrhage and necrosis, as well as anal swelling, were observed in diseased fish. At necropsy, clear fluid accumulated in the abdominal cavity, and stomach distension was evident. The liver exhibited congestion with uneven discoloration; the spleen and kidneys were congested and swollen, and intestinal inflammation was present (Figure 2).

### 3.2. Pathological Tissue Analysis

**Figure 3:**
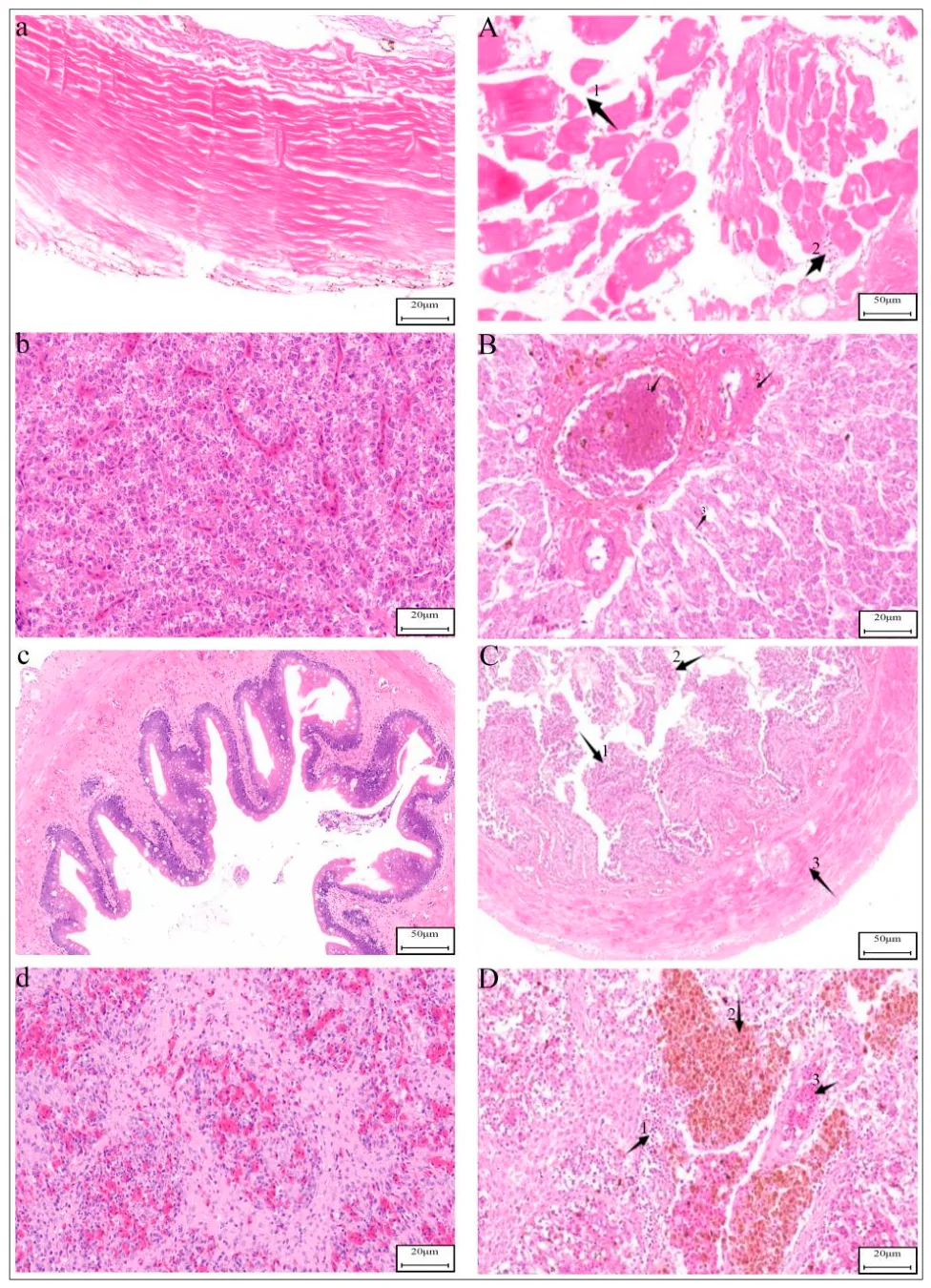
HE staining and sectioning of yellow catfish tissues to reveal pathological changes. (**a**) Muscles of healthy experimental fish; (**b**) liver of healthy experimental fish; (**c**) intestine of healthy experimental fish; (**d**) spleen of healthy experimental fish; (**A**) muscle of diseased experimental fish, (**B**) liver of diseased experimental fish; (**C**) intestine of diseased experimental fish; (**D**) spleen of diseased experimental fish. Among them, (**A**) diseased fish muscle (arrow 1 indicating muscle fiber necrosis and dissolution and arrow 2 indicating hemorrhage in the muscle space); (**B**) diseased fish liver (arrow 3 indicating cell necrosis and vacuolar degeneration and arrows 1 and 2 indicating infiltration of inflammatory cells, such as lymphocytes and macrophages, and hemorrhage); (**C**) diseased fish intestine (arrows 1 and 2 indicating necrosis and shedding of intestinal villi and arrow 3 indicating inflammation and swelling of the intestinal wall, with infiltration of inflammatory cells such as lymphocytes and macrophages); (**D**) the spleen of diseased fish (arrow 1 indicating spleen congestion and hemorrhage; arrow 2 indicating aggregation of spleen melanin macrophages and necrosis of lymphatic tissue; and arrow 3 indicating infiltration of inflammatory cells, such as lymphocytes).

Compared with tissue sections from healthy yellow catfish, the muscular lesions of diseased yellow catfish displayed muscle fiber necrosis, lysis and interstitial hemorrhage (Figure 3A). Liver tissues exhibited hepatocellular swelling, focal hepatocyte necrosis with vacuolar degeneration, massive infiltration of inflammatory cells including lymphocytes and macrophages, and extensive hemorrhage (Figure 3B). The intestinal villi underwent severe necrosis and exfoliation; the intestinal wall was infiltrated by lymphocytes, macrophages and other inflammatory cells, which caused intestinal inflammatory edema (Figure 3C). The spleen presented congestion and hemorrhage, accumulation of melanomacrophages, lymphoid tissue necrosis, as well as infiltration of lymphocytes and other inflammatory cells (Figure 3D).

### 3.3. Morphological Observation of Pathogenic Bacteria

**Figure 4:**
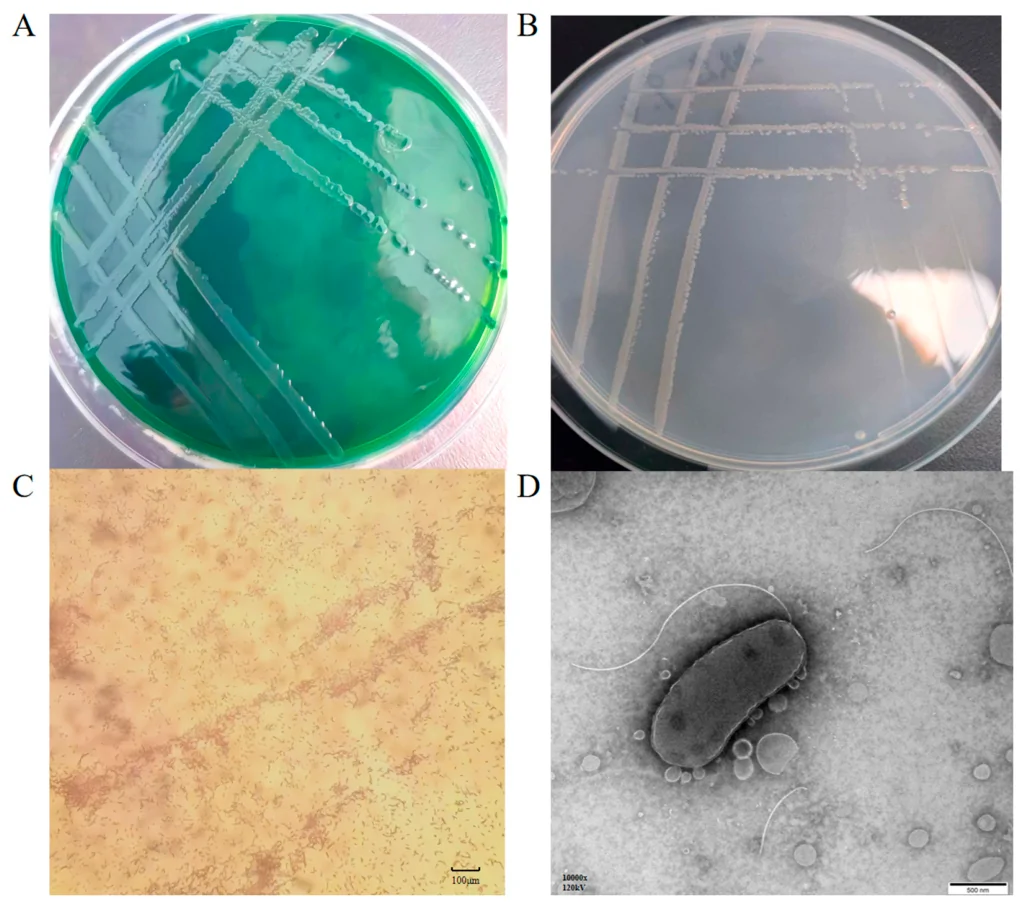
Morphological observation results of isolated strains. (**A**) Morphological characteristics of the colony in TCBS agar medium. (**B**) Morphological characteristics of the colony in TSA medium. (**C**) Gram staining results under 10 × 100 magnification. (**D**) Morphological characteristics of the strain under transmission electron microscopy.

The strain obtained through isolation is referred to as *V.zc*. is a pathogenic strain isolated and purified in this study, which was briefly named for subsequent analytical convenience). On TCBS agar medium, it forms a round colony with a smooth surface, slightly raised, neatly edged, milky white color and a green center (Figure 4A); on the TSA medium, moist, well-defined, light yellow circular colonies were formed (Figure 4B). After Gram staining, red curved short bacilli were observed under a 10 × 100 magnification oil immersion lens, further confirming that the bacteria are Gram-negative (Figure 4C). Transmission electron microscopy revealed polar single flagella and curved short bacilli (Figure 4D).

### 3.4. Artificial Infection Results

This strain exhibited high virulence to yellow catfish, with an LD_50_ value of 1.72 × 10^5^ CFU via intraperitoneal injection. The cumulative mortality rates of the intraperitoneal injection groups and negative control group are listed in Table 2. Mortality first emerged in the high-dose group two to three days post-challenge. Affected fish displayed abdominal distension, anal erythema and swelling, eroded or incomplete fins and hemorrhage at the fin bases, while cutaneous ulcerative lesions had not yet developed. The peak of morbidity and mortality occurred from day 3 to day 7 post-challenge. Infected yellow catfish presented discolored cutaneous macules, extensive severe skin ulceration and hemorrhage, abdominal distension, serous effusion in the coelom and anal swelling and hyperemia. Internal lesions included hepatic congestion, splenic and renal congestion and swelling, which closely resembled the pathological signs observed in naturally diseased fish. No typical ulcerative lesions or mortality were recorded in the control group throughout the observation period. Bacteria were isolated from artificially infected moribund yellow catfish, and the isolates were identified as *Vibrio mimicus* through colony morphology, biochemical profiling and sequence analysis of the *16S* rRNA and *vmh* genes.

### 3.5. Homology Analysis of 16S rRNA and Vmh Gene Sequences

**Figure 5:**
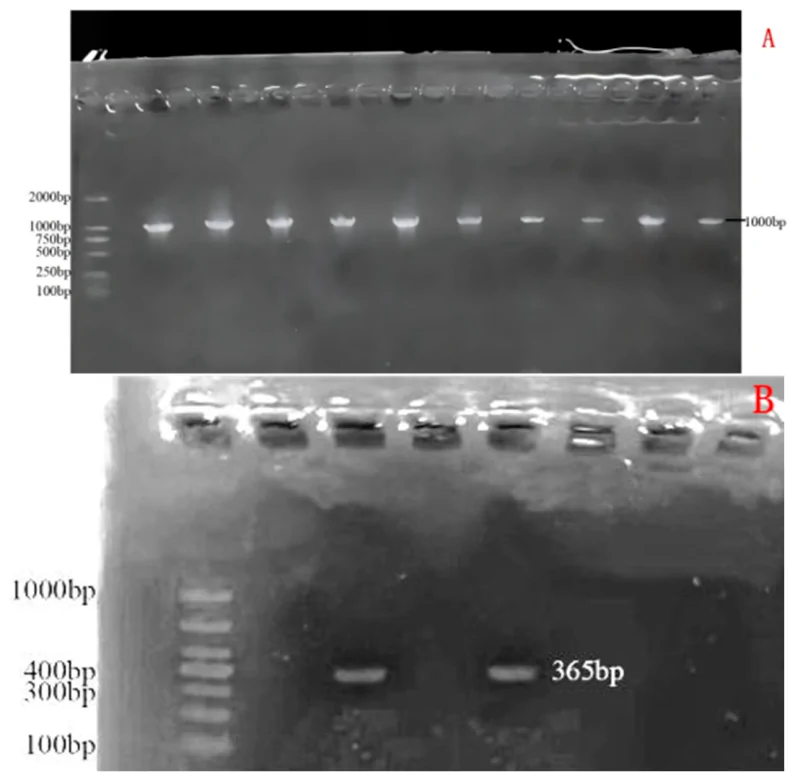
(**A**) *16s*rRNA agarose gel electrophoresis results; (**B**) vmh gene agarose gel electrophoresis results.

**Figure 6:**
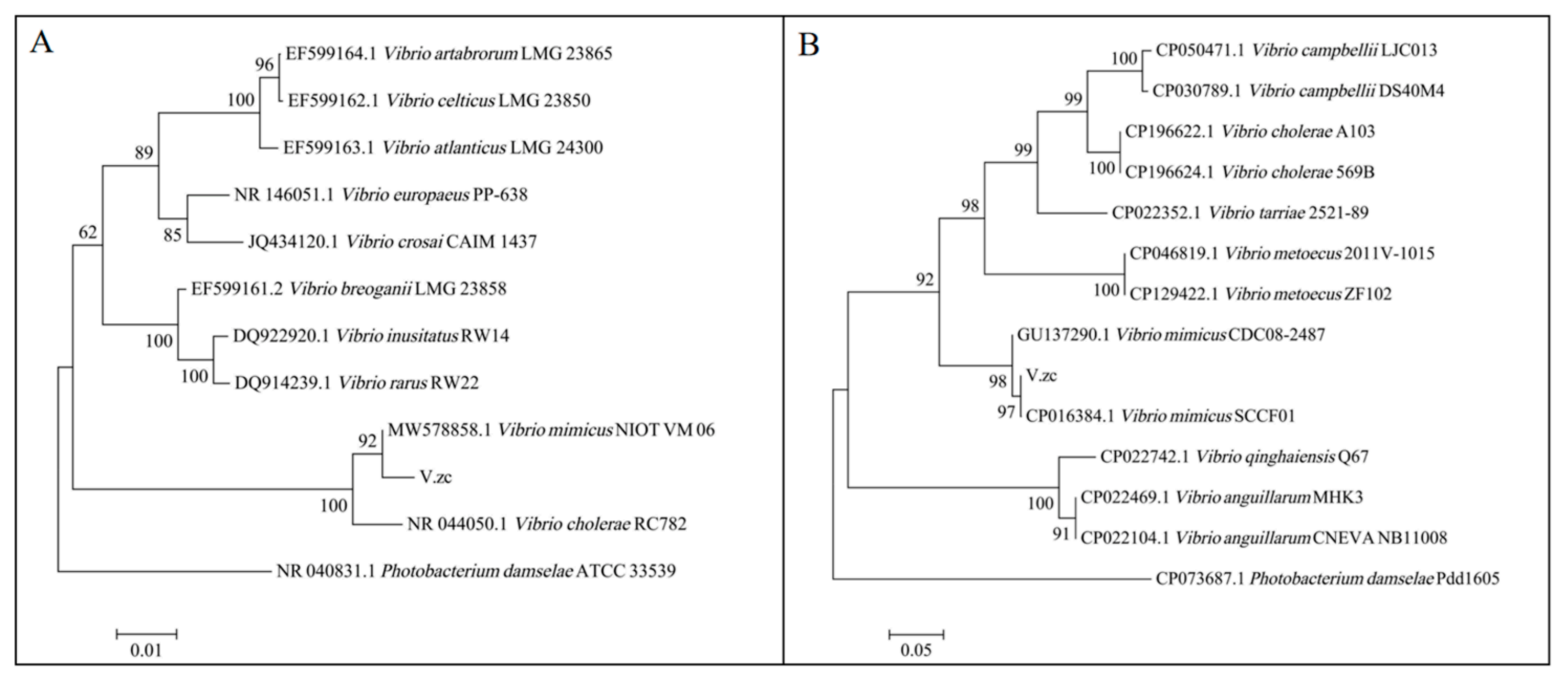
(**A**) Phylogenetic tree based on the *16S* rRNA gene sequence of the isolated strain; (**B**) phylogenetic tree based on the *Vmh* sequence of the isolated strain.

The sequencing results yielded fragments of 1000 bp and 365 bp in length, respectively (Figure 5). A phylogenetic tree was constructed based on the *16S* rRNA gene sequence (Figure 6A). The isolate (strain *V. zc*) clustered with *Vibrio mimicus* and shared high sequence homology with *Vibrio cholerae*. In the phylogenetic tree generated from the *vmh* gene sequences (Figure 6B), strain *V. zc* grouped with *Vibrio mimicus* with 97% sequence homology, whereas *Vibrio cholerae* formed a separate clade. A comprehensive homology analysis of the *16S* rRNA and vmh gene sequences confirmed that the isolated strain was identified as *Vibrio mimicus*.

### 3.6. The Impact of Temperature and pH on the Proliferation of This Strain

**Figure 7:**
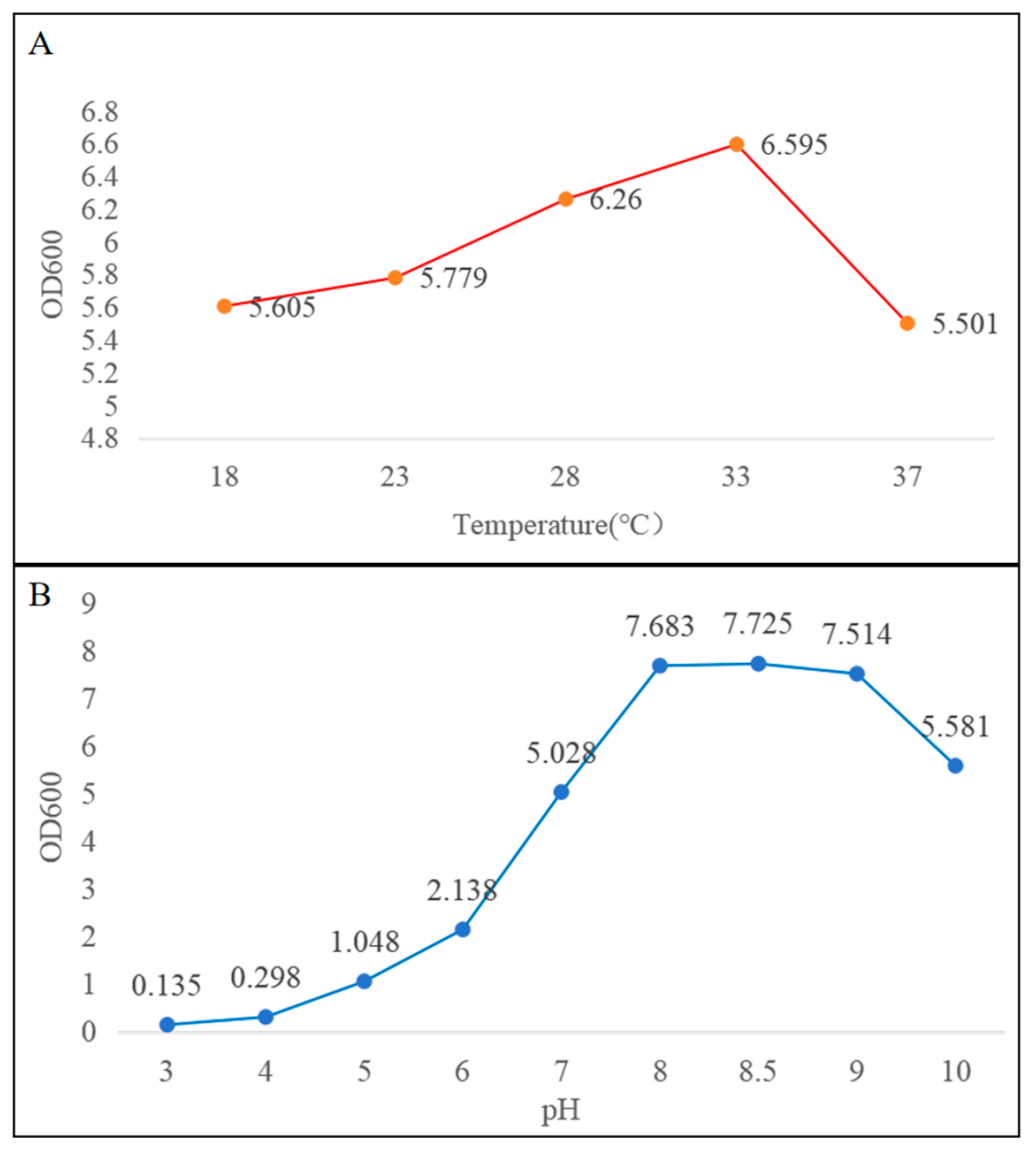
(**A**) The impact of different temperatures on the growth of this strain; (**B**) the impact of different pH levels on the growth of this strain.

After incubation for 24 h at different temperatures, absorbance values rose as temperature increased, demonstrating a positive correlation between temperature and bacterial proliferation. The strain exhibited optimal proliferation at approximately 33 °C, and growth declined at temperatures higher than 33 °C (Figure 7A).

After 24 h of incubation under varying pH conditions, absorbance increased with elevated pH. Proliferation accelerated rapidly at pH 6–8, plateaued once pH exceeded 8, and then decreased markedly. Maximum growth was observed at pH 8.5, showing that the strain grew best at this pH, followed by a gradual reduction in proliferation as the pH further increased (Figure 7B).

### 3.7. Analysis of Genome Sequencing Results

**Figure 8:**
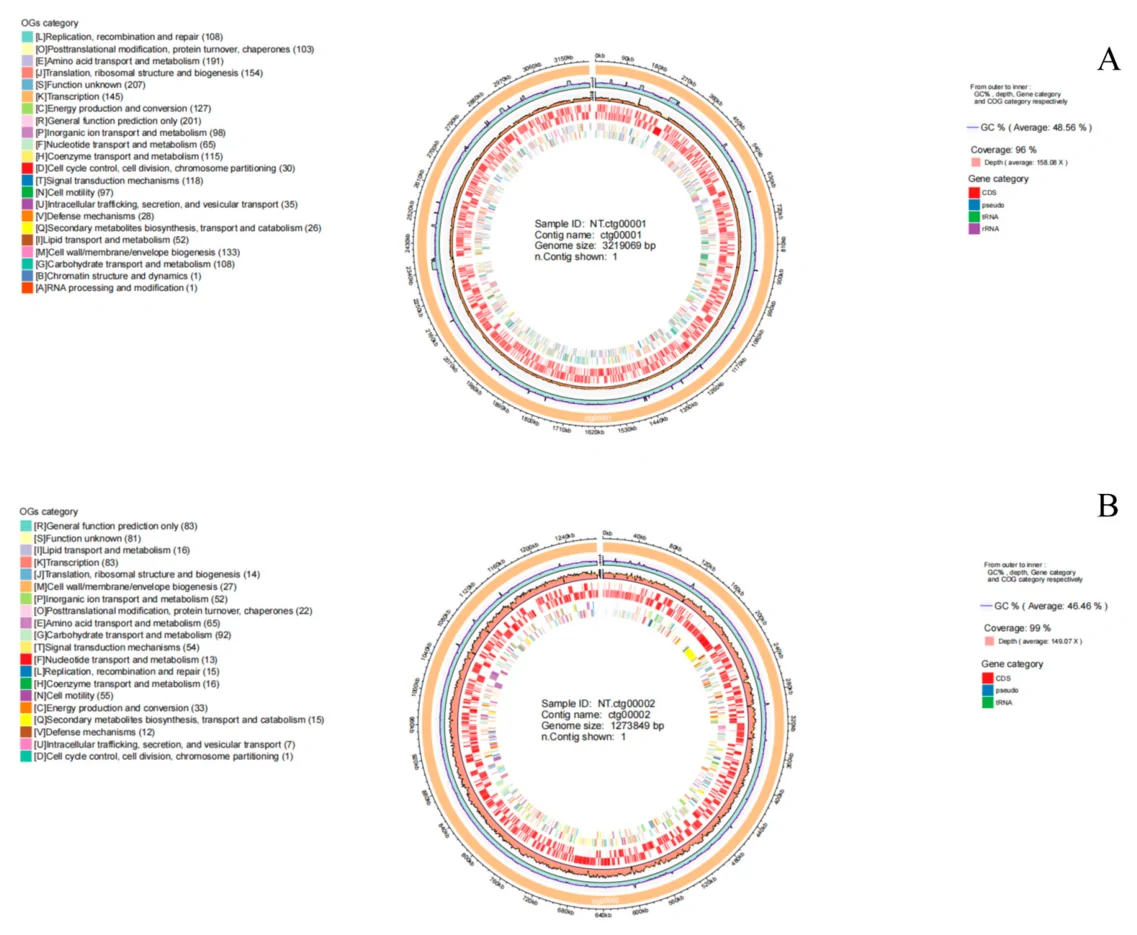
(**A**) Genomic analysis results of chromosome 1 (ctg00001) in *V.zc*; (**B**) genomic analysis results of chromosome 2 (ctg00002) in *V.zc*.

The analysis leans toward the interpretation that strain *V. zc* carries two chromosomes. For the first chromosome of *V. zc* (Figure 8A), the data record the complete genome size of this chromosome at approximately 3,219,069 bp, with an estimated GC content of 48.56% and a high sequencing coverage of 96%. Notably, 191 genes putatively associated with amino acid transport and metabolism were identified, which offers a plausible explanation for the strain’s nutrient uptake capacity to sustain growth and reproduction under specific environmental conditions. Furthermore, 145 transcription-associated genes were annotated, circumstantial evidence lending credence to their functional relevance in regulating gene expression. This chromosome also harbors abundant genes linked to cell wall, membrane and envelope biogenesis (133 genes in total), a feature that is suggestive of their presumptive role in maintaining cellular structure and normal physiological activities. A total of 28 genes for defense mechanisms were also detected, a finding that raises the possibility that this chromosome contributes to tolerance against external stressors and competing microorganisms. On balance, these observations support the notion that chromosome ctg00001 plays core roles in governing physiological processes and shaping the environmental adaptability of this Vibrio isolate.

For the second chromosome of the isolate (Figure 8B), the sequencing data yielded a genome size of roughly 1,273,849 bp, with a GC content of 46% and a sequencing coverage of 99%. Functional categorization of genes on this chromosome revealed seemingly broad functional diversity and complexity. A total of 83 transcription-related genes were annotated; by inference, this chromosome bears substantial relevance to transcriptional regulation and mediates the isolate’s responses to external environmental cues. Additionally, 16 genes assigned to lipid transport and metabolism, along with 33 genes governing energy production and conversion, point to flexible and diversified metabolic pathways conferring adaptive advantages to this Vibrio strain. Importantly, 54 genes involved in signal transduction mechanisms were also annotated on this chromosome. The presence of these genes creates a scenario whereby coordinated cellular responses to variable external environments support cell survival and propagation to a certain extent. Collectively, chromosome ctg00002 provides complementary functional modules for the ecological adaptation system of this Vibrio strain, offering a tentative mechanistic framework accounting for the organism’s survival within complex habitat environments.

**Figure 9:**
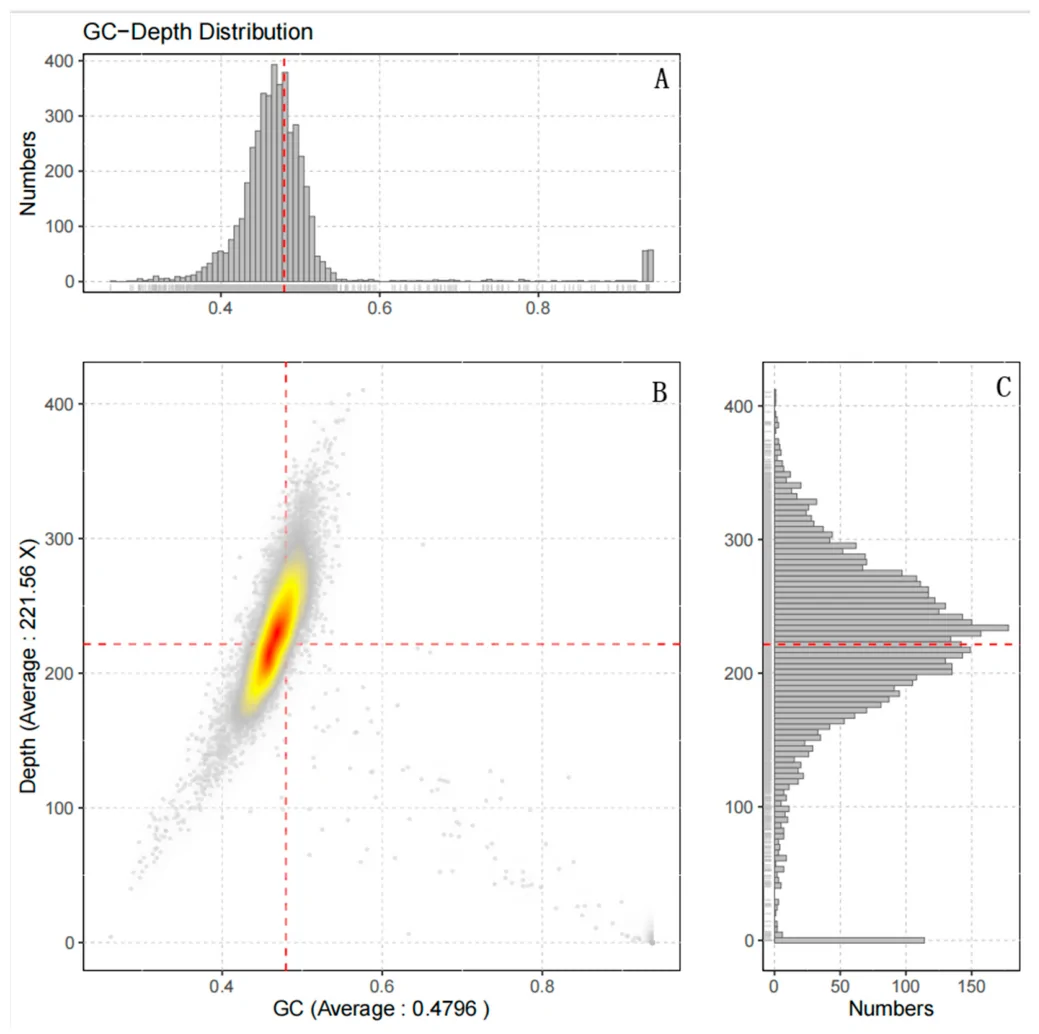
(**A**) GC content distribution plot; (**B**) GC-depth scatter plot; (**C**) depth distribution plot.

The histogram (Figure 9A) indicates that the average GC content of *V. zc* reaches 0.4796. GC values predominantly aggregate within the range of 0.4–0.6, displaying a pattern approximating a normal distribution. This distributional trait lends circumstantial evidence to the existence of base composition bias across the Vibrio genome, a feature bearing potential relevance to the strain’s ecological adaptability and evolutionary trajectory. Data points in the scatter plot (Figure 9B) aggregate within a region featuring a moderately gentle slope, a trend suggestive of a mild positive correlation between GC content and sequencing depth. These graphical patterns support a tentative interpretation that genomic segments with elevated GC values tend to produce comparatively higher sequencing coverage, a trend plausibly tied to the transcriptional activity of functional genes residing within GC-dense genomic intervals. The sequencing depth distribution graph (Figure 9C) shows that the majority of genomic regions harbor sequencing depths falling between 200× and 300×, a range broadly matching the preset targets for high-depth whole-genome sequencing. This graphical outcome provides indirect validation that the genomic datasets acquired herein carry strong potential robustness, which renders the sequences largely appropriate for downstream bioinformatic mining and functional gene annotation workflows.

Through the VFDB gene database, the possible virulence factor genes were predicted, and the gene protein sequences were compared with the VFDB by BLAST (Table 3). Through the card database, the possible drug resistance genes were predicted, and the gene protein sequences were compared with the card database by BLAST (Table 4)

Multiple genomic islands were annotated across the two chromosomes, distinguishable from core genomic sequences based on shifted GC content, skewed codon usage and flanking mobile genetic elements encoding transposases or integrases. Combined sequence signatures render these regions plausible candidates for pathogenicity islands (PAIs), which hypothetically arose from ancient horizontal gene transfer episodes. Genomic islands harbor accessory gene clusters putatively linked to virulence, which could broaden the strain’s invasive potential, capacity to evade serum immune clearance and metabolic flexibility. Distinct from stably maintained core virulence genes, island-borne genetic factors are presumed to serve auxiliary regulatory roles. Such elements may confer enhanced survival fitness within host tissues and potentially exacerbate pathological lesions throughout the infection process.

Virulence genes retrieved from the VFDB annotation are putatively responsible for encoding a multifaceted suite of pathogenic determinants. This repertoire putatively encompasses adhesins that facilitate preliminary host colonization and mucosal adhesion, secreted cytotoxins and hemolysins capable of impairing host cellular integrity and tissue barriers, siderophore biosynthesis machineries presumed to assist iron sequestration under nutrient-deficient host niches, as well as type III/VI secretion system (T3SS/T6SS) subunits that hypothetically translocate effector proteins into the host cytosol to interfere with host immune responses. The scattered localization of these virulence factors on both chromosomes lends circumstantial support to a coordinated pathogenic regulatory model. Chromosome ctg00001 predominantly encodes core virulence apparatus and housekeeping factors presumed to underpin the initiation of infection, while chromosome ctg00002 accommodates accessory virulence loci alongside signal transduction machinery (54 annotated signal transduction genes). Such gene sets are hypothesized to modulate host–pathogen crosstalk and grant the strain capacity to adjust rapidly to variable host microenvironments. Moreover, annotated genomic islands featuring abnormal GC composition, unique codon usage skews and flanking mobile genetic elements distinct from core genomic sequences qualify as plausible pathogenicity island (PAI) candidates originating from ancestral horizontal gene transfer (HGT) episodes. These discrete genomic segments carry genetic elements that may confer elevated invasive capacity, serum immune evasion or expanded metabolic flexibility, a trait hypothesized to support bacterial survival and propagation inside host tissues. Candidate HGT-associated genes, commonly adjacent to transposases, integrases or prophage-derived sequences, provide indirect evidence of genomic plasticity. This observation raises the prospect that *V. zc* has assimilated exogenous gene fragments over evolutionary time to broaden its virulence arsenal and antibiotic resistance spectrum (consistent with resistance determinants predicted via the CARD). Taken comprehensively, the coexistence of horizontally acquired virulence loci and chromosomally encoded transcriptional regulators (274 transcription-associated genes distributed over the two chromosomes) points toward a complex, tiered virulence control network. This molecular framework is conjectured to allow *V. zc* to dynamically adjust its pathogenic phenotype in response to host environmental signals, which in turn may aid colonization, tissue penetration and sustained persistent infection in susceptible fish hosts.

### 3.8. Drug Sensitivity Test Results

The susceptibility testing results of this strain against 15 common antibacterial agents covering nine categories are summarized in Table 5. The isolate exhibited high susceptibility to penicillin, piperacillin, kanamycin, ceftriaxone, cefoperazone, levofloxacin, norfloxacin, roxithromycin, azithromycin, furazolidone and tetracycline. It displayed intermediate susceptibility to gentamicin and ofloxacin, as well as resistance to sulfamethoxazole and clindamycin.

## 4. Discussion

*Vibrio mimicus* was first described as a biochemically atypical *Vibrio cholerae* [20]. *Vibrio mimicus* exists in many natural environments and has become a significant human pathogen [21]. Its clinical symptoms include fever, diarrhea, nausea, vomiting and abdominal cramps. The primary transmission routes of *Vibrio mimicus* infection are the ingestion of raw shellfish or contact with wounds [14]. A new systemic ulcer disease associated with *Vibrio mimicus* infection in cultured *Pelteobagrus fulvidraco* has been reported. The disease spread rapidly, with a cumulative mortality rate reaching 70% to 100%, severely hindering the development of yellow catfish farming. This was the first report that *Vibrio mimicus* could cause severe disease in freshwater fish [22], but such reports are still very scarce. In recent years, continuous outbreaks of *V. mimicus*-induced ulcer syndrome have been recorded in freshwater *Siluriformes* aquaculture across southern China, further confirming the emerging threat of this pathogen to freshwater fish breeding industry [8]. According to statistics, the disease is common in the rainy season from April to May and the high-temperature period from August to October every year, with May to June and August to September representing high-risk periods, consistent with the seasonal distribution pattern of *V. mimicus* in subtropical freshwater farms reported by Elgendy et al. [23]. This disease mainly affects 3–5 cm fish, especially in the fry stage, with high mortality. Effective prevention and control of this disease have become a focus in yellow catfish farming. Several other Vibrio species can also cause ulcer syndrome in aquatic animals [24,25,26,27].

This study characterized the typical severe regular cutaneous ulcers observed in farmed *Pelteobagrus fulvidraco*. The diseased fish displayed severe hemorrhagic muscular necrosis and perianal erythema and swelling. Postmortem dissection revealed abdominal effusion and gastric distension; internal organ lesions included hepatic congestion, congestive swelling of the spleen and kidney and intestinal inflammation. Histopathological comparisons with healthy yellow catfish tissues further uncovered distinct pathological lesions in affected individuals: ulcerated musculature with necrotic, lysed muscle fibers and interstitial hemorrhage; swollen hepatocytes with focal necrosis and vacuolar degeneration, accompanied by massive infiltration of lymphocytes, macrophages and severe hemorrhage; extensive necrosis and shedding of intestinal villi, with inflammatory cell infiltration triggering intestinal wall edema; splenic congestion and hemorrhage, accumulation of melanomacrophages, lymphoid tissue necrosis and diffuse lymphocyte infiltration. These clinical and histopathological manifestations were highly consistent with those documented in prior studies [6,22]. Integrated transcriptomic research further verified that such multi-organ necrotic inflammatory lesions originate from uncontrolled cytokine storms and tissue remodeling pathways triggered by *V. mimicus* infection in yellow catfish [1].

Morphological examination and genomic identification collectively verified that the causative pathogen was *Vibrio mimicus*. Owing to their close phylogenetic relatedness, *V. mimicus* and *V. cholerae* share numerous physiological and biochemical traits, making phenotypic differentiation difficult [28]. Distinct phenotypic correlations between colony morphology on TCBS agar and sucrose metabolic capacity have been well documented across *Vibrionaceae* species in previous bacteriological studies [29]. TCBS medium functions as a classic selective-differential substrate relying on bromothymol blue–thymol blue pH indicators to discriminate between sucrose-fermenting and non-sucrose-fermenting vibrios; acid metabolites generated during sucrose utilization lower the microenvironmental pH surrounding colonies and shift indicator color to yellow. Accordingly, strains capable of sucrose metabolism generally develop large, smooth, opaque yellow colonies with intact margins after 18–24 h of incubation. By contrast, isolates deficient in sucrose fermentation fail to accumulate sufficient acidic byproducts, maintaining neutral-to-alkaline local pH and forming green or teal colonies that tend to exhibit smaller diameters and thinner translucent peripheral zones, a phenotypic pattern widely accepted as a presumptive marker for sucrose-negative vibrios such as *V. parahaemolyticus*, *V. vulnificus* and *V. mimicus* [30,31]. The two species display nearly 100% sequence identity in the *16S* rRNA gene and approximately 80% DNA–DNA hybridization relatedness [32]. Genomic analyses have established hemolysin as a key virulence determinant of *V. mimicus*, which exerts hemolytic and enterotoxic effects by disrupting erythrocyte membranes and elevating intracellular cAMP levels in intestinal epithelial cells [33]. Two hemolysin variants have been identified in *V. mimicus*, namely thermostable and thermolabile hemolysins, of which the thermolabile hemolysin encoded by the vmh gene is ubiquitously distributed. Previous surveys have confirmed the universal presence of the vmh gene across all clinical and environmental *V. mimicus* isolates [34,35].

In the present work, phylogenetic reconstruction based on *16S* rRNA sequences grouped our isolate *V.zc* within the *V. mimicus* clade with 99% sequence homology, yet this strain also shared high sequence similarity with *V. cholerae*. To overcome the limitation of species discrimination relying solely on the non-protein-coding *16S* rRNA gene [36], we supplemented molecular identification with vmh gene sequencing. The *vmh* sequence of strain *V.zc* exhibited 99% homology to reference *V. mimicus* strains, whereas its identity to *V. cholerae* fell below 92%. Consistently, the vmh-derived phylogenetic tree clustered strain *V.zc* exclusively with *V. mimicus*, clearly separating it from *V. cholerae*. This molecular evidence definitively identified strain *V.zc* as *Vibrio mimicus*.

Bioinformatic prediction identified multiple genomic island regions distributed on both chromosomes of strain *V.zc*, which could be distinguished from core genomic sequences by altered GC content, biased codon usage and flanking mobile genetic element-coding genes (transposase and integrase). According to sequence features alone, these regions were preliminarily screened as potential pathogenicity island candidates, which may have originated from historical horizontal gene transfer events. It should be emphasized that all such judgements are derived solely from genomic sequence signatures, without corresponding functional verification experiments. The accessory gene clusters inside predicted genomic islands were annotated as being associated with virulence via database alignment; we speculate that these genes might participate in regulating bacterial invasion, immune escape or environmental metabolism, but there is no direct evidence to confirm that these island-encoded genes can independently enhance the pathogenicity of this strain or produce strain-specific lesion phenotypes in *Pelteobagrus fulvidraco*. Unlike stably inherited core virulence genes, genes located on genomic islands may play auxiliary regulatory roles, yet their actual contribution to in vivo bacterial survival and tissue damage remains unclear.

By exploring the effects of temperature and pH on the in vitro proliferation of *Vibrio mimicus*, we have drawn the following conclusions: When the temperature was 23–33 °C, the growth. was positively correlated with temperature. The higher the temperature, the greater the proliferation, with the highest proliferation observed at 33 °C; above 33 °C, proliferation began to decline. With increasing pH, the proliferation effect increased significantly, with a rapid increase from pH 6 to 8. After the pH reached 8, proliferation entered a plateau stage, and the proliferation rate decreased considerably, reaching a peak at pH = 8.5. This indicates that the bacteria has the best proliferation effect at pH8.5, and then it begins to decline with increasing pH. The results of this study are similar to those reported previously [18]. Climate-associated environmental monitoring of aquatic *Vibrio* further supported the finding that warm water temperatures and weakly alkaline water pH are two core abiotic factors that boost the proliferation and epidemic risk of *V. mimicus* in freshwater ponds [37]. The use of antibiotics has played a positive role in controlling bacterial diseases in aquatic animals. However, the extensive use of antibiotics has led to negative impacts such as microbial resistance [38], food residues and environmental pollution, which cannot be ignored. The results of antimicrobial susceptibility tests showed that this strain of *V. mimicus* is highly sensitive to most commonly used antibiotics, providing data to support for the prevention and control of *V. mimicus*. The artificial regression infection test proved the pathogenic potential of *Vibrio mimicus* isolate (*v.zc*), which exhibited high virulence to *Pelteobagrus fulvidraco*. The LD50 value obtained by intraperitoneal injection of different concentrations of bacterial suspension was 1.72 × 105 CFU. The experimental data showed that the injection of higher concentrations of bacterial suspension could cause a large number of deaths among experimental fish in a short time, with mortality occurring much more rapidly than the development of symptoms. At lower concentrations of the bacterial suspension, deaths occurred at different times during a test cycle, and the same symptoms occurred. These symptoms were highly similar to those of the diseased fish identified at the beginning of the experiment. The pathogenic bacterium *Vibrio mimicus* could also be isolated and purified from the infected fish. The results of this experiment provide a scientific basis for the isolation, identification and analysis of *xanthobagrus fulvidraco* canker and *Vibrio mimicus*. We also conducted immersion and oral challenges in artificial infection experiments. Nevertheless, obvious pathogenicity was not observed via these two approaches, and the corresponding data were excluded from this study. For this reason, intraperitoneal injection was adopted as the only infection route. This model primarily evaluates systemic virulence rather than natural infectivity, and the obtained LD_50_ value is valid only for intraperitoneal injection challenge. This constitutes a major limitation of our study. In future investigations, we will further explore alternative infection routes to complement our results and generate more comprehensive reference data.

Notably, all functional roles of the above virulence-associated genes, PAIs and horizontally transferred genetic elements are merely bioinformatic predictions deduced from genomic annotation. Direct in vivo and in vitro experimental validation remains indispensable for determining the exact contributions of these candidate virulence factors to the infectivity, tissue damage and host adaptability of *V. zc*. Subsequent functional assays, including gene knockout mutants, infection challenge tests and protein expression characterization, are required to corroborate the bioinformatic inferences and clarify their authentic pathogenic functions.

## Figures and Tables

**Table 1:** Primers and sequences.

Type	Primer	Sequence
universal primers	27F	AGA GTT TGA TCC TGG CTC AG
	1492R	CGG TTA CCT TGT TAC GAC TT
specific primers	vmh-F	GGT AGC CAT CAG TCT TAT CAC G
	vmh-R	ATC GTG TCC CAA TAC TTC ACC G

**Table 2:** Total cumulative mortality recorded for yellow catfish after injection.

Bacterial Suspension Concentration	Accumulative Death/Total (Individual)					Mortality	LD50 (CFU/Fish)
	1 d	3 d	5 d	7 d	10 d		
1.5 × 10^8^	0/10	7/10	10/10	10/10	10/10	100%	1.72 × 10^5^
1.5 × 10^7^	0/10	4/10	7/10	9/10	9/10	90%	
1.5 × 10^6^	0/10	0/10	6/10	6/10	6/10	60%	
1.5 × 10^5^	0/10	0/10	5/10	5/10	5/10	50%	
1.5 × 10^4^	0/10	0/10	2/10	3/10	3/10	30%	
1.5 × 10^3^	0/10	0/10	1/10	1/10	1/10	10%	
control	0/10	0/10	0/10	0/10	0/10	0%	/

Note: the control group was injected with the same volume of normal saline.

**Table 3:** Virulence factor.

VFclass	Virulence Factors	Related Genes
Toxin	Accessory cholera enterotoxin	Ace
	Hemolysin/cytolysin	vvhA
	Thermolabile hemolysin	tlh
	Thermostable direct hemolysin	tdh
	*V. mimicus* hemolysin	vmh
	Zonula occludens toxin	zot
	Cholera toxin	ctxA
		ctxB
Adherence	Toxin-coregulated pilus (type IVB pilus)	tcpA
		tcpB
		tcpC
Endotoxin	LPS	bplB
		bplC
		bplF
Secretion system	T3SS1	tyeA
		vcrD
		vscA

**Table 4:** Drug resistance gene.

Gene	Resistance	Coverage (%)
CRP	fluoroquinolone; macrolide; penam	100
msbA	nitroimidazole	83.3
Vibrio_cholerae_varG	carbapenem	98.55
tet(35)	tetracycline	88.38
ugd	peptide	98.11
catB9	phenicol	68.89

**Table 5:** Drug sensitivity results.

Antibiotics		Concentration (ug/Piece)	Diameter of Inhibition (mm)	Result
Penicillins	Penicillin	30	22	S
	piperacillin	100	38	S
Sulfonamides	Bactrim	25	0	R
Aminoglycosides	Kanamycin	30	22	S
	Gentamicin	10	19	I
Cephalosporins	Cefoperazone	30	41	S
	Levofloxacin	75	36	S
Quinolones	Levofloxacin	5	21	S
	Norfloxacin	10	24	S
	Ofloxacin	5	19	I
Macrolides	Roxithromycin	15	25	S
	Azithromycin	15	30	S
Nitroimidazoles	Furazolidone	30	28	S
Tetracyclines	Tetracycline	30	33	S
Lincosamides	Clindamycin	2	0	R

Note: S—sensitivity, I—moderate sensitivity, R—resistance; S ≥ 20 mm; I: 10~20 mm; R ≤ 10 mm.

## Data Availability

The data presented in this study are openly available in [GenBank] at [https://www.ncbi.nlm.nih.gov], reference number [PZ812459; SAMN62346783].

## Abbreviations

TSA	Tryptone soy agar
H&E	hematoxylin and eosin
PCR	polymerase chain reaction
NJ	neighbor-joining method
*16S*rRNA	*16S* ribosomal RN
LD50	median lethal dose
LB	(Luria-Bertani) medium is a basic bacterial culture medium commonly used for the pre-cultivation of bacteria to facilitate their rapid proliferation for experimental requirements. It contains tryptone, yeast extract and sodium chloride to supply nitrogen, carbon, vitamins and inorganic ions for bacterial growth.
